# Disclosing suboptimal indications for emergency caesarean sections due to fetal distress and prolonged labor: a multicenter cross-sectional study at 12 public hospitals in Nepal

**DOI:** 10.1186/s12978-020-01039-x

**Published:** 2020-12-17

**Authors:** Helena Litorp, Rejina Gurung, Mats Målqvist, Ashish Kc

**Affiliations:** 1 Department of Women’s and Children’s Health, Uppsala University, Uppsala, Sweden; 2 Department of Global Public Health, Karolinska Institutet, Stockholm, Sweden; 3 Golden Community, Jawagal, Lalitpur, Nepal; 4 Society of Public Health Physician’s Nepal, Kathmandu, Nepal

**Keywords:** Caesarean section, Indications, Quality of care, Fetal distress, Prolonged labour, Low-income countries, Nepal

## Abstract

**Background:**

Global caesarean section (CS) rates have raised concern of a potential overuse of the procedure in both high- and low-resource settings. We sought to assess management and outcomes of deliveries with emergency CSs due to fetal distress and prolonged labor at 12 public hospitals in Nepal and determine factors associated with suboptimal CS indications.

**Methods:**

We conducted a cross-sectional study on all deliveries between the 14th of April 2017 and the 17th of October 2018 at 12 public hospitals in Nepal and included all emergency CSs due to fetal distress and prolonged labor. Analysis was conducted using Pearson chi-square test and bivariate and multivariate logistic regression.

**Results:**

The total cohort included 104,322 deliveries of which 18,964 (18%) were CSs (13,095 [13%] emergency CSs and 5230 [5.0%] elective CSs). We identified 1806 emergency CSs due to fetal distress and 1322 emergency CSs due to prolonged labor. Among CSs due to fetal distress, only 36% had fetal heart rate monitoring performed according to protocol, and among CSs due to prolonged labor, the partograph was completely filled in only 8.6%. Gestational age < 37 weeks and birth weight < 2500 g were associated with more suboptimal CS indications due to fetal distress (adjusted odds ratio [aOR] 1.4, 95% confidence interval [CI] 1.1–1.8 and aOR 1.7, 95% CI 1.3–2.2 respectively) than those with gestational age > 37 weeks and birth weight > 2500 g. We found no association between suboptimal CS indications and maternal ethnicity or education level.

**Conclusions:**

As fetal heart rate monitoring and partograph are fundamental to diagnose fetal distress and prolonged labor, the inappropriate monitoring proceeding CS decisions disclosed in our study indicate that CSs were performed on suboptimal indications. We call for improved quality of intrapartum monitoring, enhanced documentation in medical records, and structured auditing of CS indications in order to curb the potentially harmful CS trend.

## Background

Although emergency caesarean section (CS) can be a life-saving intervention when complications occur during labour, global CS rates have raised concerns of a potential overuse, i.e. CSs performed without medically robust indications, in both high- and low-resource settings [1–4]. In an attempt to identify a minimum CS rate for resource-poor contexts, covering the prevalence of the six most common life-threatening conditions for mother and fetus, Belizán et al. suggest a facility-based CS rate of 9% to indicate a rational use of CS in such settings [2]. In recent years, the South East Asian region has demonstrated among the world’s largest absolute increases in CS rates from 4.4 to 19.2% [1], and Nepal specifically has been highlighted as one of the countries with the highest increase in CS rates among the richest fifth of the population [3].

Fetal distress and prolonged labor are repeatedly reported as the most common reasons for emergency CSs, but are often associated with suboptimal diagnosis and management [4–14]. In order to make accurate and timely emergency CS decisions, health care providers need to assess the fetal heart rate and monitor the progress of labour using a partograph [4]. Both fetal heart rate monitoring (FHRM) and partograph have shown potential to decrease perinatal deaths in low-resource settings [15–17]. Although there is no evidence on what intervals FHRM should be performed in order to obtain optimal perinatal outcomes [18, 19], the World Health Organization (WHO) and the International Federation of Gynaecology and Obstetrics recommend that fetal heart rate should be assessed every 15–30 min during the first stage of active labour and every 5 min during the second stage of labour [19, 20]. The evidence whether partograph use improves maternal and perinatal outcomes is inconclusive [21], however, the WHO recommends that progress of labour should be monitored every 4 h during the active stage of labour and subsequently recorded in a partograph [22, 23]. Nevertheless, despite evidence supporting FHRM and partograph use in low-resource settings, many health care providers in developing countries fail to adhere to these recommendations [11, 24–28].

Nepal is a low-income country that, despite a number of social challenges and still struggling to build up its midwifery workforce [29], has made impressive improvements in maternal and child outcomes during the last decades [30]. The under-5-mortality rate has declined from 91/1000 live births in 2001 to 38/1000 live births in 2014 [31] and the maternal mortality ratio has declined from 553/100,000 live births in 2000 to 186/100,000 live births in 2017 [32]. Although only 69% of women undergo the recommended number of four or more antenatal care visits, 84% of women attend antenatal care at least once during their pregnancy and 58% of births are assisted by a skilled birth professional [33]. Of the total births in Nepal in 2016, CSs accounted for 9%, but with large discrepancies depending on women’s educational level (5% among women with no education vs. 20% among women with secondary level education), wealth (2% among women in the lowest wealth quantile vs. 28% in the highest), and type of facility (12% in public facilities vs. 35% in private) [33]. Hence, many researchers argue that there might be an overuse of CS in some facilities in Nepal and that this needs to be investigated in further detail [9, 34, 35].

Most previous research to assess accuracy of CS indications, in Nepal as well as other low-resource settings, have been based at one facility and included relatively small sample sizes [5, 6, 8, 12, 34]. It has been reported that the majority of stillbirths and cases of neonatal encephalopathy in Nepal are associated with intrapartum hypoxia and suboptimal care [36], and that there is a lack of proper FHRM and partograph use in Nepali hospitals [27]. In light of this evidence and the current global discourse on CS overuse in low-resource settings, we sought to assess management and outcomes of CSs performed due to fetal distress and prolonged labour at 12 public hospitals in Nepal, and determine factors associated with suboptimal CS indications.

## Methods

With the aim of assessing management and outcomes of CSs performed due to fetal distress and prolonged labour, we conducted an observational, cross-sectional study using data collected on all deliveries between the 14th of April 2017 and the 17th of October 2018 at 12 public hospitals in Nepal. The study was part of a large stepped-wedged randomized controlled trial conducted to evaluate the effect of a quality improvement intervention on perinatal care in Nepal (NePeriQIP), targeting specifically the quality of neonatal resuscitation of non-breathing newborns [37, 38].

### Setting

Included hospitals had between 1000 and 11,000 deliveries per year, were government-funded referral centers for maternal and newborn care and provided comprehensive emergency obstetric care. With a total number of deliveries of about 100,000 in the 12 hospitals, the NePeriQIP data collection accounted for almost 29% of the total health facility deliveries in Nepal during the study period. Despite all of them mostly being in the flatlands, the hospitals were different in terms of service coverage and diverse in relation to ethnicity, language, and religion. Mid-Western Regional and Seti Zonal Hospitals were located in the most disadvantaged regions in Nepal in terms of literacy, access to services, and life expectancy. Bheri Zonal Hospital in Nepalgunj had a large number of minority Muslim communities, while in Bharatpur, some of the most fringe communities came for maternal and sick newborn services. The labour unit in each hospital was led by registered nurses with 2 months additional skilled birth attendant training, hence capable of managing normal pregnancies as well as assessing and referring complicated ones. FHRM was performed using Pinard fetoscope or hand-held Doppler. In the study context, most women with one or several previous CSs routinely underwent elective CSs, if not presenting in labour before their scheduled operation, in which case they would undergo emergency CS. In the high-volume hospitals there were specialized sick newborn units led by pediatricians, while in the low-volume hospitals, sick newborns were managed by medical doctors at the pediatric units.

### Participants

We included all deliveries conducted through emergency CS due to fetal distress or prolonged labour among women consenting to participate in the NePeriQIP study [37, 38]. Elective CSs, defined as CSs performed before onset of active stage of labour as recorded in the medical record and hospital registers, were excluded from the analyses.

### Data collection and management

A data surveillance system was established in all the hospitals to collect data on deliveries [37, 38]. For obstetric variables, data were extracted from the Maternity Registers and medical records by trained data collectors using a data retrieval form. For sociodemographic variables, data were collected by the same data collectors through semi-structured interviews with mothers before discharge. Completed forms were then assessed by a data coordinator at the hospital for completeness and accuracy before being entered digitally into the data base by the data entry and management team. For entering and cleaning data, we used the Census and Survey Processing System (CSPro).

### Variables and outcomes

We included the following indications as representing emergency CSs due to fetal distress: “fetal distress”, “fetal tachycardia”, “fetal bradycardia”, “meconium stained liquor” (including thick, medium, and thin), “decreased fetal movements”, “abnormal fetal movements”, “cord around the neck”, and “severe birth asphyxia”. As the diagnosis of prolonged labour, obstructed labour, and cephalo-pelvic disproportion is often unspecific and substandard [5, 10, 12, 14, 28] in many low-resource settings, we included the following indications to represent emergency CSs due to prolonged labour: “prolonged labour, “obstructed labour”, “cephalo-pelvic disproportion”, “non-progress of labour”, “big baby”, “macrosomia”, “poor maternal effort”, “less maternal effort”, “short stature”, “protracted labour”, “protracted pelvis”, “irregular pelvis”, “high head”, “protracted dilatation”, and “protracted descent”.

In the NePeriQIP data collection, data was collected on FHRM during labour and was categorized as “as per protocol” (defined as FHRM performed every 30 min according to the local standards), “sporadically, but more than once”, “only once”, and “not done”. We determined suboptimal indication of emergency CS due to fetal distress to be FHRM performed only once or not at all. Partograph use was categorized as “completely filled” (defined as cervical dilatation and descent of fetal head recorded at least every four hours), “partially filled”, or “not filled”, and we determined suboptimal indication for emergency CS due to prolonged labour to be no filled partograph.

### Statistical analysis

The cleaned data were exported into Statistical Package for Social Sciences (SPSS) version 25 for analysis. We used descriptive statistics to present socio-economic and obstetric characteristics of women undergoing CS due to fetal distress and prolonged labour. For CSs performed due to fetal distress, we calculated the proportion monitored in terms of fetal heart rate, the proportion of fetuses with abnormal fetal heart rate 15 min before birth, the proportion of newborns receiving bag-and-mask ventilation, the proportion of newborns with Apgar score < 7 at 5 min, and the proportion of neonatal first-day mortality. For CSs performed due to prolonged labour, we calculated the proportion that was monitored with partograph and the proportion that received augmentation of labour with oxytocin, as we wanted to assess what interventions had been undertaken to accomplish a vaginal delivery. Proportions of FHRM, perinatal outcomes, partograph use, and augmentation with oxytocin were compared with the total cohort using the Pearson chi-square test. *p*-values below 0.05 were considered statistically significant.

Hypothesizing that suboptimal CS indications would be associated with socio-economic status and obstetric characteristics [33], we conducted bivariate logistic regression to calculate the crude odds ratio (cOR) and 95% confidence intervals (CI) for the likelihood of suboptimal CS indications among women with induced labour (yes vs. no), gestational age < 37 weeks (yes vs. no), disadvantaged ethnic group (comprising Dalit, Muslim and other disadvantaged groups vs. advantageous group comprising Brahmin, Chettri, Madeshi, and Janjati), no education (illiterate vs. literate, basic education and above), and birth weight < 2500 g (yes vs. no). Ethnic group was chosen as it reflected the woman’s social status within the Nepali social class system. Missing values were excluded from the analysis. Variables with statistically significant associations with the outcome under investigation were included in multivariate logistic regression analyses to calculate the adjusted odds ratio (aOR).

### Ethical consideration

Written informed consent was obtained from the mothers before inclusion in the NePeriQIP study and confidentiality was guaranteed. The study was approved by Ethical Review Board of Nepal Health Research Council (reference number 26-2017). For the current study, the researchers worked with a data base coded as per the patient’s identity.

## Results

A total of 104,223 deliveries were included in the NePeriQIP data collection, of which 18,964 (18%) were CSs. Among the CSs, 13,095 (69%) were emergency CSs, 5230 (28%) were elective CSs, and 420 (2.2%) had missing data on CS category. We identified 1806 CSs due to fetal distress, representing 14% of all emergency CSs, and 1322 CS due to prolonged labour, representing 10% of all emergency CSs. Despite scrutinizing all registered emergency CS indications and re-coding them into the categories fetal distress or prolonged labour as described in the Methods section, 7169 (55%) emergency CSs were still lacking indication (indication registered as “other”). Characteristics of women undergoing CS due to fetal distress and prolonged labour, as well as characteristics of women in the total cohort, are presented in Table 1. Compared to women in the total cohort, women undergoing CS due to fetal distress and prolonged labour more often gave birth to their first baby and were more often admitted before the start of active labour. Induction of labour was more common among women who underwent CS due to prolonged labour compared to women who underwent CS due to fetal distress and women in the total cohort. There were no large differences in terms of maternal age, education level, ethnicity, birth weight, and gestational age between women who underwent CS due to fetal distress or prolonged labour and the total cohort. There was, however, less missing data among women who delivered through CS. Characteristics and intrapartum monitoring among women with and without documented emergency CS indication are presented in Additional file 1.

**Table 1:** Characteristics of deliveries with emergency caesarean section (CS) due to fetal distress and prolonged labour

Characteristics	CS due to fetal distress N = 1806	CS due to prolonged labour N = 1322	Total cohort N = 104,223
Maternal age, mean (SD)	24.1 (4.2)	24.3 (4.4)	24.0 (4.3)
Parity			
0-para	1,023 (57%)	797 (60%)	40,538 (39%)
1 previous birth	491 (27%)	304 (23%)	31,365 (30%)
2–5 previous births	292 (16%)	292 (17%)	17,416 (17%)
Missing	0	1 (0.1%)	14,893 (14%)
Education level			
Illiterate	47 (2.6%)	37 (2.8%)	3134 (3.0%)
Literate	118 (6.5%)	71 (5.4%)	6528 (6.3%)
Basic education	188 (10%)	125 (9.5%)	11,298 (11%)
Secondary and above	696 (39%)	437 (33%)	41,710 (40%)
Higher	148 (8.2%)	212 (16%)	5657 (5.4%)
Missing	609 (34%)	440 (13%)	35,896 (34%)
Ethnicity ^a^			
Advantaged groups	922 (51%)	627 (47%)	52,772 (51%)
Disadvantaged groups	884 (49%)	695 (53%)	51,031 (49%)
Missing	0	0	420 (0.4%)
Pregnancy induced hypertensive disorders	14 (0.8%)	10 (0.8%)	267 (0.3%)
Antepartum hemorrhage	1 (0.1%)	1 (0.1%)	95 (0.1%)
Stage of labour on admission			
Not in labour	522 (40%)	706 (39%)	19,783 (19%)
Latent phase of first stage of labour	698 (53%)	964 (53%)	40,781 (39%)
Active phase of first stage of labour	94 (7.1%)	129 (7.1%)	23,045 (22%)
Second stage of labour	8 (0.6%)	7 (0.4%)	3877 (3.7%)
Missing	0	0	16,234 (16%)
Induction of labour			
With prostaglandins	236 (13%)	264 (20%)	14,890 (14%)
With amniotomy	54 (3.0%)	42 (3.2%)	2046 (2.0%)
With oxytocin	154 (8.5%)	161 (12%)	7434 (7.1%)
No induction	1362 (76%)	855 (65%)	53,627 (52%)
Missing	0	0	16,324 (16%)
Mode of delivery			
Spontaneous vaginal	0	0	64,846 (62%)
Instrumental vaginal	0	0	3001 (2.9%)
Caesarean section	1806 (100%)	1322 (100%)	18,964 (18%)
Assisted breech	0	0	437 (0.5%)
Missing	0	0	16,939 (16%)
Birth weight			
< 2500 g	269 (15%)	160 (12%)	14,945 (14%)
≥ 2500 g	1366 (76%)	971 (73%)	66,732 (64%)
Missing	171 (9.5%)	191 (14%)	22,546 (22%)
Gestational age			
< 37 weeks	220 (17%)	306 (17%)	15,665 (15%)
≥ 37 weeks	911 (74%)	1329 (69%)	66,012 (63%)
Missing	171 (9.5%)	191 (14%)	22,546 (22%)

^a^Advantageous group representing Brahmin, Chettri, Madeshi, and Janjati. Disadvantaged group representing Dalit, Muslim and other disadvantaged groups

Among CSs performed due to fetal distress, 36% had FHRM performed according to protocol during labour and 15% had no FHRM registered in their medical records (Table 2). In half of the CSs due to fetal distress, no fetal heart rate was checked 15 min before delivery and in the other half, fetal heart rate was registered as normal. In terms of outcomes, 0.4% of newborns delivered by CSs due to fetal distress required bag-and-mask ventilation compared to 0.8% in the total cohort (*p* = 0.02). The percentage of babies with Apgar score < 7 at 5 min was 4.9% among CSs due to fetal distress, which was in line with the 4.2% in the total cohort (*p* = 0.17), and there was no statistically significant difference in neonatal first-day mortality (*p* = 0.06).

**Table 2:** Monitoring and perinatal outcomes among women delivered through caesarean section (CS) due to fetal distress

Monitoring and outcomes	CS due to fetal distress N = 1806	Total cohort N = 104, 223	*p*-value
Fetal heart rate monitoring during labour			
Yes, as per protocol	647 (36%)	38,677 (37%)	
Yes, sporadically (> once)	559 (31%)	23,352 (22%)	
Yes, once	327 (18%)	18,897 (18%)	
No	273 (15%)	7,063 (7%)	
Missing	0	16,234 (16%)	< 0.001
Fetal heart rate 15 min before delivery			
Normal	796 (44%)	45,839 (44%)	
Abnormal	3 (0.2%)	46 (0%)	
Absent	1 (0.1%)	72 (0.1%)	
Not recorded	1,006 (56%)	42,032 (40%)	
Missing	0	16,234 (16%)	< 0.001
Bag-and-mask ventilation	7 (0.4%)	822 (0.8%)	0.02
Apgar < 7 at 5 min	88 (4.9%)	3726 (4.2%)	0.17
Neonatal first-day mortality	1 (0.1%)	307 (0.3%)	0.06

Among women who underwent CS due to prolonged labour, the partograph was completely filled in 8.6% of deliveries (Table 3) and in 69%, no partograph was filled. Only 35% of women who subsequently underwent CS due to prolonged labour had received augmentation of labour with oxytocin. The corresponding figures for the total cohort were 23% completely filled partograph, 38% with no filled partograph (*p* < 0.001), and 29% augmentation of labour (*p* = 0.46).

**Table 3:** Monitoring and management among women delivered through caesarean section (CS) due to prolonged labour

Monitoring and management	CS due to prolonged labour N = 1322	Total cohort N = 104,223	*p*-value
Partograph use			
Yes, completely filled	114 (8.6%)	23,994 (23%)	
Yes, partially filled	303 (23%)	24,375 (23%)	
Not filled	905 (69%)	39,620 (38%)	
Missing	0	16,234 (16%)	< 0.001
Augmentation of labour with oxytocin			
Yes	459 (35%)	29,711 (29%)	
No	863 (65%)	58,278 (56%)	
Missing	0	16,234 (16%)	0.46

A total of 600 (33%) CSs due to fetal distress had a suboptimal CS indication, i.e. no FHRM or FHRM performed only once. Factors associated with a suboptimal fetal distress indication were gestational age < 37 weeks (cOR 1.4, 95% CI 1.1–1.8) and birth weight < 2500 g (cOR 1.7, 95% CI 1.3–2.3) (Table 4), which remained statistically significant in the multivariate analyses adjusting for induction of labour (aOR 1.4, 95% CI 1.1–1.8, and aOR 1.7, 95% CI 1.3–2.2, respectively). Induction of labour was associated with a lower odds of suboptimal fetal distress indication (cOR 0.69, 95% CI 0.53–0.88), also after adjusting for gestational age < 37 weeks and birth weight < 2500 g (aOR 0.70, 95% CI 0.54–0.92). Among CSs performed due to prolonged labour, 905 (69%) were categorized as being performed on suboptimal indications, i.e. had no filled partograph. In contrast to FHRM, deliveries with gestational age < 37 weeks (cOR 0.56, 95% CI 0.41–0.75) and those with birth weight < 2500 g (cOR 0.43, 95% CI 0.30–0.60) had more often been monitored with partograph than those with gestational age > 37 weeks and babies weighing > 2500 g at birth. We found no statistically significant associations between suboptimal CS indications due to fetal distress or prolonged labour and socioeconomic factors such as maternal ethnicity or education level, hence these variables were not included in the multivariate model.

**Table 4:** Bivariate logistic regression of obstetric and social factors and suboptimal caesarean section (CS) indications

Obstetric and social factors	CS due to fetal distress N = 1806			CS due to prolonged labour N = 1322		
	FHRM as per protocol or > once	No FHRM or FHRM only once	cOR (95% CI) for suboptimal indication	Partograph completely or partially filled	Partograph not filled	cOR (95% CI) for suboptimal indication
Induction of labour						
Yes	285 (73%)	105 (27%)	0.69 (0.53–0.88)	143 (34%)	282 (66%)	0.87 (0.68–1.1)
No	921 (65%)	425 (35%)	1	274 (31%)	623 (70%)	1
Gestational age						
< 37 weeks	185 (61%)	121 (41%)	1.4 (1.1–1.8)	96 (44%)	124 (56%)	0.56 (0.41–0.75)
> 37 weeks	912 (69%)	417 (31%)	1	274 (30%)	637 (70%)	1
Ethnicity						
Disadvantaged groups	587 (66%)	297 (34%)	1.0 (0.85–1.26)	223 (32%)	472 (68%)	0.95 (0.75–2.1)
Advantaged groups	619 (67%)	303 (33%)	1	194 (31%)	433 (69%)	1
Education level						
Illiterate	35 (75%)	12 (26%)	0.69 (0.35–1.3)	13 (35%)	24 (65%)	0.99 (0.50–2.0)
Literate, incl. basic education and higher	766 (67%)	384 (33%)	1	294 (35%)	551 (65%)	1
Birth weight						
< 2500 g	152 (57%)	117 (44%)	1.7 (1.3–2.3)	80 (50%)	80 (50%)	0.43 (0.30–0.60)
> 2500 g	945 (69%)	421 (31%)	1	290 (30%)	681 (70%)	1

## Discussion

In this large-scale observational study from Nepal covering almost a third of the country’s facility births during the time period, we found that only 36% of CSs performed due to fetal distress were monitored according to protocol in terms of fetal heart rate and in 15% no FHRM was performed. Babies born through CS due to fetal distress less often required bag-and-mask ventilation compared to babies in the total cohort, but we found no differences in the proportion of newborns with Apgar score < 7 at 5 min and first-day mortality. Among CSs performed due to prolonged labour, only 8.6% had a completely filled partograph and only 35% received augmentation of labour with oxytocin. Women who had gestational age < 37 weeks and birth weight < 2500 g had more often a suboptimal indication for CS due to fetal distress, but we found no association between suboptimal CS indications and maternal ethnicity or education level.

As reported in other studies from low- and middle-income countries [14, 27, 31, 39], the use of FHRM and partograph in our study was far from meeting both local and international standards, and although FHRM and partograph are essential to diagnose fetal distress and prolonged labour, women had, as reported elsewhere [35], undergone CS on such indications without proper proceeding monitoring. In line with a small, cross-sectional study from India [7], we found that perinatal outcomes after CS due to fetal distress were similar to those in the total cohort in terms of Apgar score and first-day mortality, and that bag-and-mask ventilation after birth was even less common than in the total cohort, reflecting either an overuse of CS due to fetal distress performed in healthy fetuses, or that CSs were made in time to avert adverse perinatal outcomes. Although oxytocin to augment labour is a key interventions to manage prolonged labour, only 35% of women who underwent CS due to prolonged labour had received this treatment, in line with other studies from Tanzania reporting CSs performed due to prolonged labour despite the lack of other, less invasive, interventions than surgery [5, 10, 11, 14]. Our study also reports important shortfalls in terms of documentation, as more than half of CSs lacked a documented indication.

Fetuses with gestational age < 37 weeks and birth weight < 2500 g comprise an especially vulnerable group in terms of developing birth asphyxia, hence proper FHRM is crucial to prevent adverse events. In our study, however, CS due to fetal distress was more often performed without proper proceeding FHRM among pregnancies with gestational age < 37 weeks and birth weight < 2500 g. Although we can only speculate on reasons for this, one possible explanation is that care professionals, being aware of the increased risk of birth asphyxia during labour, were more liberal with CSs due to fetal distress in this group even when they had not monitored the fetal heart rate as per protocol. On the other hand, surprisingly, women who underwent CS due to prolonged labour and had gestational age < 37 weeks or low birth weight were more often monitored with partograph than those with term pregnancies and normal birth weight. Our study provides no explanation of these findings, but practical and local circumstances might have affected the use of partograph, as reported elsewhere [25]. In contrast to other studies, in which a concern of medically doubtful CSs have been reported among women with higher socio-economic status [9, 34, 35], we found no association between suboptimal CS indications and ethnicity or education level.

In order to avert potentially harmful short- and long-term consequences of unnecessary CSs in low-resource settings [40–42], emergency CS indications need to be as accurate as possible and rely on proper proceeding monitoring. A recent systematic review of 37 studies from low-and middle-income countries concludes that low-cost equipment such as Pinard fetoscope, wind-up Doppler, and partograph, preferably combined with clinical information such as meconium stained liquor and fetal scalp stimulation, has the potential to improve outcomes and avoid unnecessary CSs in low-resource settings, hence improving care does not require expensive, high-technology investments [16]. A major threat to timely and accurate FHRM and partograph use is, however, that these measures highly depend on human resources, and many low-resource settings struggle with a shortage of staff, lack of training, and substandard guidelines [16, 25]. In order to enhance intrapartum monitoring, local and contextual barriers and incentives for FHRM and partograph use need to be addressed [25]. Previous literature report evidence that supportive organizational policy, senior leadership, and staff involvement might promote partograph use [25], and a recent study from Ethiopia demonstrated increased use of partograph among staff who had received on-the-job training in obstetric care, rather than only their pre-service training [26]. In India, an intervention at 44 public health facilities including orientation training of doctors and program managers, assessment, and feedback improved the use of FHRM [43], and in a university hospital in Tanzania, criteria-based audit improved the accuracy of diagnosis of fetal distress and prolonged labour among women who underwent CS [5, 6]. Developing and implementing low-cost technologies such as the Moyo fetal heart rate monitor to enhance monitoring, unburden midwives, improve detection of abnormal fetal heart rate, and increase the well-being of mothers [44–48], might be another way to tackle this problem in low-resource settings, and such an intervention is currently implemented in eight hospitals in Nepal by our research team [49].

The main strength of our study is the large sample size collected at 12 district hospitals distributed all over the country, making our results generalizable and applicable to other health care facilities in low-resource settings. The data collection teams were organized to secure quality of data, and repeated validity checks were undertaken. In terms of missing data, these were kept to a minimum among women undergoing CS, probably because these women had a relatively long hospital stay which allowed for complementing gaps in the data collection form. Missing data was, however, a problem when analyzing data for the total cohort, comprising about 16%. Another limitation of the study was the well-known complexity of interpreting CS indications, as CS indications are not clearly defined [50], as raised previously in this paper. Several indications might also be registered for the same operation, requiring a hierarchical structure [50]. In our study, we interpreted and categorized CS indications retrospectively without availability of the original medical records, which made interpretations more difficult, moreover, more than half of the emergency CSs lacked a documented indications (indication registered as “other”). Therefore, we most likely missed CSs that were performed, but not recorded, as CSs due to fetal distress and prolonged labour, making our sample size smaller. Lastly, our dataset did not provide information on results of FHRM or what the partograph displayed, but only the frequency of monitoring, hence indications might have been suboptimal although frequency of monitoring was as per standards.

## Conclusion

As FHRM and partograph are fundamental to diagnose fetal distress and prolonged labour, the substandard monitoring proceeding CSs in the hospitals included in our study indicate that CSs were performed on suboptimal indications. Moreover, our study highlights the need for better standardization and documentation of CS indications, as almost half of CSs lacked a registered indication. Given the large sample size, including 12 public hospitals in Nepal together comprising almost a third of the country’s facility births, we believe our results to be generalizable and applicable to many other low- and middle-income countries. In order to curb the potentially harmful trend of CS overuse in low-resource settings, we call for improved quality of intrapartum monitoring, structured auditing of CS indications at facility level and implementation of clearer diagnostic criteria of fetal distress and prolonged labour.

## Supplementary information

**Additional file 1.** Characteristics and intrapartum monitoring among women with and without documented emergency caesarean section (CS) indication.

## Data Availability

The data sets analyzed for the current study are available from the corresponding author on reasonable request.

## Abbreviations

CI: Confidence intervals
CS: Caesarean section
FHRM: Fetal heart rate monitoring
NePeriQIP: Nepal perinatal quality improvement project
cOR: Crude odds ratio
aOR: Adjusted odds ratio
WHO: World Health Organization
