# Supplementary material for: Disclosing suboptimal indications for emergency caesarean sections due to fetal distress and prolonged labor: a multicenter cross-sectional study at 12 public hospitals in Nepal
Source: Reprod Health. 2020 Dec 17;17:197. doi: 10.1186/s12978-020-01039-x (PMC7745386; doi:10.1186/s12978-020-01039-x)
Supplement: Supplementary file 1 — Additional file 1. Characteristics and intrapartum monitoring among women with and without documented emergency caesarean section (CS) indication. [file 12978_2020_1039_MOESM1_ESM.docx]

*Characteristics and intrapartum monitoring among women with and without documented emergency caesarean section (CS) indication.*

| **Characteristics** | **No documented CS indication**  **N= 7,169** | **Documented CS indication**  **N= 5,926** |
| --- | --- | --- |
| **Maternal age, mean (SD)** | 24.6 (4.5) | 24.7 (4.4) |
| **Parity** |  |  |
| 0-para | 3,798 (53%) | 2,722 (46%) |
| 1 previous birth | 2,019 (28%) | 2,001 (34%) |
| 2-5 previous births | 1,352 (19%) | 1,202 (20%) |
| Missing | 0 | 1 (0%) |
| **Education level** |  |  |
| Illiterate | 282 (3.9%) | 150 (2.5%) |
| Literate | 537 (7.5%) | 344 (5.8%) |
| Basic education | 812 (11%) | 634 (11%) |
| Secondary and above | 3,908 (55%) | 1,969 (33%) |
| Higher | 93 (1.3%) | 706 (12%) |
| Missing | 1,537 (21%) | 2,123 (36%) |
| **Ethnicity** |  |  |
| Advantaged groups | 3,681 (51%) | 3,048 (51%) |
| Disadvantaged groups | 3,488 (49%) | 2,878 (49%) |
| Missing | 0 | 0 |
| **Stage of labour on admission** |  |  |
| Not in labour | 3,771 (53%) | 2,808 (47%) |
| Latent phase of first stage of labour | 2,832 (40%) | 2,699 (46%) |
| Active phase of first stage of labour | 533 (7.4%) | 385 (6.5%) |
| Second stage of labour | 33 (0.5%) | 34 (0.6%) |
| Missing | 0 | 0 |
| **Birth weight** |  |  |
| < 2,500 g | 937 (13%) | 1,027 (17%) |
| ≥ 2,500 g | 5,875 (82%) | 4,2221 (71%) |
| Missing | 357 (5.0%) | 678 (11%) |
| **Gestational age** |  |  |
| < 37 weeks | 836 (12%) | 1,178 (20%) |
| ≥ 37 weeks | 5,976 (83%) | 4,070 (69%) |
| Missing | 537 (5.0%) | 678 (11%) |
| **Hospital** |  |  |
| Hospital 1 | 993 (14%) | 861 (15%) |
| Hospital 2 | 283 (3.9%) | 484 (8.2%) |
| Hospital 3 | 30 (0.4%) | 25 (0.4%) |
| Hospital 4 | 1,550 (22%) | 1,698 (29%) |
| Hospital 5 | 1,180 (17%) | 726 (12%) |
| Hospital 6 | 50 (0.7%) | 75 (1.3%) |
| Hospital 7 | 818 (11%) | 767 (13%) |
| Hospital 8 | 526 (7.3%) | 402 (6.8%) |
| Hospital 9 | 7 (0.1%) | 9 (0.2%) |
| Hospital 10 | 607 (8.5%) | 300 (5.1%) |
| Hospital 11 | 977 (14%) | 412 (7.0%) |
| Hospital 12 | 148 (2.1%) | 167 (2.8%) |
| Missing | 0 | 0 |
| **Fetal heart rate monitoring during labour** |  |  |
| Yes, as per protocol | 2,790 (39%) | 1,945 (33%) |
| Yes, sporadically (> once) | 1,834 (26%) | 1,732 (29%) |
| Yes, once | 1,588 (22%) | 1,030 (17%) |
| No | 957 (13%) | 1,219 (21%) |
| Missing | 0 | 0 |
| **Partograph use** |  |  |
| Yes, completely filled | 781 (11%) | 415 (7.0%) |
| Yes, partially filled | 1,293 (18%) | 997 (17%) |
| Not filled | 5,095 (71%) | 4,514 (76%) |
| Missing | 0 | 0 |
